# Heterologous Expression of GbTCP4, a Class II TCP Transcription Factor, Regulates Trichome Formation and Root Hair Development in Arabidopsis

**DOI:** 10.3390/genes10090726

**Published:** 2019-09-19

**Authors:** Yi Wang, Yuehua Yu, Quanjia Chen, Guanghong Bai, Wenwei Gao, Yanying Qu, Zhiyong Ni

**Affiliations:** College of Agronomy, Xinjiang Agricultural University, Urumqi 830052, China; wangyi604987664@126.com (Y.W.); yuyuehua1213@sina.com (Y.Y.); chqjia@126.com (Q.C.); bgh601@126.com (G.B.); gww0911@163.com (W.G.); xjyyq5322@126.com (Y.Q.)

**Keywords:** sea-island cotton, TCP transcription factor, trichome formation, root hair development

## Abstract

Two class I family teosinte branched1/cycloidea/proliferating cell factor1 (TCP) proteins from allotetraploid cotton are involved in cotton fiber cell differentiation and elongation and root hair development. However, the biological function of most class II TCP proteins is unclear. This study sought to reveal the characteristics and functions of the sea-island cotton class II TCP gene *GbTCP4* by biochemical, genetic, and molecular biology methods. GbTCP4 protein localizes to nuclei, binding two types of TCP-binding *cis*-acting elements, including the one in its promoter. Expression pattern analysis revealed that *GbTCP4* is widely expressed in tissues, with the highest level in flowers. *GbTCP4* is expressed at different fiber development stages and has high transcription in fibers beginning at 5 days post anthesis (DPA). *GbTCP4* overexpression increases primary root hair length and density and leaf and stem trichomes in transgenic *Arabidopsis* relative to wild-type plants (WT). GbTCP4 binds directly to the *CAPRICE* (*CPC*) promoter, increasing *CPC* transcript levels in roots and reducing them in leaves. Compared with WT plants, lignin content in the stems of transgenic *Arabidopsis* overexpressing *GbTCP4* increased, and *AtCAD5* gene transcript levels increased. These results suggest that GbTCP4 regulates trichome formation and root hair development in *Arabidopsis* and may be a candidate gene for regulating cotton fiber elongation.

## 1. Introduction

Cotton is one of the most important fiber crops worldwide. Cotton fiber quality directly affects the market competitiveness of textiles [1]. Cotton fibers are unicellular trichomes derived from ovule epidermal cells. Fiber development can be divided into four distinct yet overlapping stages: initiation, cell elongation, secondary cell wall biosynthesis, and maturation [1,2]. Cotton fiber quality is measured in terms of length and strength, which mainly depend on the cell elongation and secondary cell wall biosynthesis growth stages [3]. Upland cotton (*Gossypium hirsutum* L.) and sea-island cotton (*G. barbadense* L.), which are widely grown, are allotetraploid cultivars [4]. Upland cotton has wide adaptability and a high yield, accounting for 99% of the raw cotton produced in China [5]. Sea-island cotton fiber is superior to upland cotton in length, fineness, and strength [3]. Exploring the molecular mechanisms involved in fiber development in sea-island cotton can provide useful information to generate superior fiber quality traits of upland cotton species through molecular breeding [6].

An increasing number of studies have shown that cotton fiber development has a similar transcriptional regulatory mechanism to that of the development of *Arabidopsis* leaf trichomes [7]. Therefore, *Arabidopsis* leaf trichomes can be used as an ideal system to study cotton fiber development genes. Teosinte branched1/cycloidea/proliferating cell factor1 (TCP) is a plant-specific protein family that has a conserved TCP domain and consists of 59 amino acid residues that form a basic helix–loop–helix (bHLH) structure [8]. The TCP domain is required for protein–protein interactions and DNA binding. According to the different TCP domains, the TCP family is divided into two subfamilies: Class I (TCP-P or PCF) and Class II (TCP-C). Class II TCP proteins are further subdivided into two subclasses, ECE (TB1/CYC) and CIN, based on differences in their amino acid domains [8]. In *Arabidopsis*, the TCP family consists of 24 members, some of which are involved in the regulation of trichome development [9,10]. For example, AtTCP4 is a class II TCP protein that inhibits trichome branching in *Arabidopsis* leaves and inflorescence stems by direct transcriptional activation of the trichome branching negative regulator GLABROUS INFLORESCENCE STEMS [9]. *AtTCP15* is highly expressed in trichomes, and compared with wild-type (WT) plants, plants in which *AtTCP15* expression is inhibited present increased trichome branch numbers [10].

To date, 36 and 38 *TCP* genes have been investigated in the diploid cotton species *G. arboreum* [11] and *G. raimondii* [12], respectively, and 74 *TCPs* have been identified in allotetraploid upland cotton [13]. Previous studies have found that two class I family TCP proteins from allotetraploid cotton are involved in cotton fiber cell differentiation and elongation and root hair development [14,15]. For instance, in sea-island cotton, *GbTCP*, the homologue of the *AtTCP15* gene, is a crucial regulator of fiber elongation and root hair development by positively regulating jasmonic acid biosynthesis and the response and other pathways, as determined using overexpression and RNA interference silencing techniques [14]. In upland cotton, *GhTCP14* is most similar to *AtTCP14*. *Arabidopsis* overexpressing *GhTCP14* showed enhanced differentiation and elongation of root hairs and trichomes. GhTCP14 regulates the differential expression of several auxin synthesis- and transport-related genes, including *IAA3*, *AUX1*, and *PIN2* [15]. However, the biological function of most class II TCP proteins in cotton is unclear. Therefore, it is important to identify class II TCP transcription factors in cotton and elucidate their functional mechanism.

Our previous study identified 75 *GbTCP* genes from sea-island cotton [16]. Real-time quantitative PCR (qPCR) results revealed that some *GbTCP* genes were expressed at different cotton fiber developmental stages, suggesting that these *GbTCP* genes may be involved in the regulation of cotton fiber development [16]. In this study, a sea-island cotton class II TCP gene, *GbTCP4*, was cloned and functionally identified. Overexpression of the *GbTCP4* gene increased root hair length, root hair and trichome density, and the lignin content in transgenic *Arabidopsis* plants relative to WT plants. *CAPRICE* (*CPC*) and *AtCAD5* are target genes of GbTCP4. These results indicate that GbTCP4, a class II TCP transcription factor, regulates trichome formation and root hair development in *Arabidopsis* and can be used as a candidate gene for cotton fiber quality improvement.

## 2. Materials and Methods

### 2.1. Plant Materials

The experimental material of this study was the sea-island cotton cultivar Xinhai 21, which was planted in the experimental field of Alar City, Xinjiang. The ovules (0 d), flowers, calyxes and receptacles were collected on the flowering day, as were the cotton fibers at 5 d, 10 d, 15 d, 20 d, 25 d, 30 d, and 35 d after flowering. The seeds of Xinhai 21 were soaked in 70% absolute ethanol, disinfected for 3 min, washed repeatedly with sterile water 3 to 5 times, spread in a germination box containing filter paper and cultured at 28 °C for 4 d. The hypocotyls were collected. The seedlings were transplanted into the nutrient solution to continue to grow, and when the first true leaves emerged, the main roots, fibrous roots, stems and leaves were collected. All samples were stored at −80 °C after liquid nitrogen freezing for RNA extraction.

Plants of the *Arabidopsis thaliana* ecotype Columbia (Col-0) were used to transform the *GbTCP4* gene. *Arabidopsis* was cultured in a greenhouse with a photoperiod of 16/8 h (day/night) and a culture temperature of 23 °C. The first two rosette leaves and roots of the transgenic and WT *Arabidopsis* were collected for analysis of the transcript level of the *CPC* gene. *Arabidopsis* stems grown for 6 weeks in nutrient soil were collected for analysis of lignin content and transcript levels of genes involved in the lignin pathway.

### 2.2. RNA Extraction and cDNA Synthesis

Total RNA was extracted, and contaminating genomic DNA was removed as previously described [17]. Total RNA (200 ng) was reverse transcribed to cDNA using RevertAid^TM^ M-MuLV Reverse Transcriptase (Fermentas, Vilnius, Lithuania).

### 2.3. Real-Time Quantitative Polymerase Chain Reaction

qPCR was performed with green qPCR Master Mix (Fermentas, Vilnius, Lithuania) [16]. The *GbUBQ7* and *AtUBQ3* genes were used as internal standards for sea-island cotton and *Arabidopsis*, respectively. Amplification was performed using an ABI 7500 Fast Real-Time PCR instrument (Applied Biosystems Inc., Foster City, CA, USA). The qPCR amplification system and amplification program followed the manufacturer’s instructions. The gene and internal reference gene cycle threshold (Ct) values of each sample were detected. Experimental data were analyzed by the 2^−ΔΔCt^ method. The experiments were performed in three biological replicates. The hypocotyls were used as control samples to analyze the transcript level of the *GbTCP4* gene in different tissues of cotton. Primer sequences are shown in Supplementary Table S1.

### 2.4. Gene Cloning

According to the transcriptome data from the ovules of the sea-island cotton cultivar Xinhai 21 [16], the *GbTCP4* gene was amplified using the first strand of the ovule cDNA as a template. The amplified *GbTCP4* gene was ligated into the pMD18-T vector (TaKaRa, Dalian, China) and sequenced to obtain the open reading frame (ORF) sequence of *GbTCP4*. Primer sequences are shown in Supplementary Table S1.

### 2.5. Bioinformatic Analysis

The molecular weight (MW), isoelectric point (pI), and homology analysis of the GbTCP4 protein were predicted using DNAMAN7 software [16]. Multiple sequence alignments were performed using ClustalX 1.83 software, and phylogenetic trees were constructed using MEGA 4.1 software [16]. The subcellular localization of GbTCP4 was predicted online using the PSORT program [16]. The 3000 bp promoter sequence upstream of *GbTCP4* and some of the known transcription factor genes involved in root hair and trichome development were obtained from the *G. barbadense* genome (https://cottonfgd.org) and The *Arabidopsis* Information Resource (TAIR, http://arabidopsis.org), respectively. The PLACE database was used for promoter sequence analyses [18].

### 2.6. Subcellular Localization

For the subcellular localization experiment, the ORF of GbTCP4 was subcloned into the plant expression vector pCAMBIA1304 containing the GFP reporter gene to generate the *35S:GbTCP4-GFP* fusion expression vector. Transient transformation of onion epidermal cells was performed by the *Agrobacterium*-mediated method. First, 1 cm^2^ of onion epidermal cells was soaked in *Agrobacterium* solution for 30 min, and the surface of the bacterial solution was filtered, transferred to 1/2 MS solid medium and incubated at 25 °C for 2 d under a 16 h light/8 h dark photoperiod. After the epidermal cells of the inoculated and co-cultured cells were washed, they were placed on a glass slide and observed under a Zeiss LSM510 confocal microscope (Zeiss, Oberkochen, Germany).

### 2.7. Transcriptional Activation Assay

For the transcriptional activation assay, the ORF sequence of *GbTCP4* was cloned into the pGBKT7 vector. The pGBKT7 and GbTCP4-pGBKT7 plasmids were transformed into AH109 yeast cells using the Frozen-EZ Yeast Transformation II Kit (Zymo Research, Orange, CA, USA). The pGBKT7 vector carries the TRP1 nutritional marker for selection in yeast. AH109 contains ADE2 and HIS3 screening markers. The transformed yeast cells were cultured on SD/-Trp medium and SD/-Trp-His-Ade medium for 30 h at 30 °C, and the growth of yeast cells was observed.

### 2.8. Yeast One-Hybrid Assay

For yeast one-hybrid assays, the ORF of *GbTCP4* was inserted into pGADT7-Rec2 (Clontech, Palo Alto, CA, USA) as the effector (GbTCP4-pGADT7). Three tandem repeats of TCP I (TGGGTCCCACAT), TCP II (TTGTGGGCCCCT), the mutated TCP I (TAGATTCTAAAG), and the mutated TCP II (TCGGGAGACTCG) were synthesized with reference to previous studies [15]; different fragments containing different TCP motifs of the promoter sequences of *AtCPC*, *AtCAD5,* and *GbTCP4* were amplified from genomic DNA. These sequences were cloned into pHIS2 (Clontech, Palo Alto, CA, USA) upstream of the reporter gene *HIS3*. Co-transformation of pHIS2 with or without the TCP *cis*-element sequences and GbTCP4-pGADT7 into Y187 cells was performed as described by Yu et al. (2017) [19]. The pGADT7 vector carries the LEU2 nutritional marker for selection in yeast. The pHIS2 vector carries the TRP1 nutritional marker for selection in yeast. The transformed yeast cells were cultured on SD/-Trp-Leu medium and SD/-Trp-His-Leu medium supplemented with 10 mM or 30 mM 3-amino-1,2,4-triazole (3-AT) for 30 h at 30 °C, and the growth of yeast cells was observed. Primer sequences are shown in Supplementary Table S1.

### 2.9. Generation of Transgenic Arabidopsis Plants

To construct a constitutively overexpressing plant expression vector driven by the cauliflower mosaic virus 35S promoter (35S CaMV), the ORF of *GbTCP4* was cloned into the pCAMBIA3301 vector (Cambia, Canberra, Australia). *Agrobacterium*-mediated *Arabidopsis* transformation was performed as described by Ni et al. (2013) [20]. Transgenic *Arabidopsis* seeds were screened using glufosinate. Three homozygous T3 generation transgenic *A. thaliana* plants were selected for further experiments.

### 2.10. Root Hair Observation and Measurement

For root hair analyses, Col-0 and *35S:GbTCP4* seeds were planted on Murashige and Skoog (MS) medium plates. When two true leaves emerged, 30 seedlings from Col-0 and *35S:GbTCP4* were placed in a new MS medium plate. After 1 week of upright growth, the root hair density and root hair length were measured at 1 cm above the main root tip. Photographs were taken under a Classica SK200 digital microscope (Motic, Xiamen, China). All experiments were repeated three times.

### 2.11. Trichome Observation and Measurement

For trichome analyses, Col-0 and *35S:GbTCP4* seeds were planted on MS medium plates. When two cotyledons grew, 30 seedlings from Col-0 and *35S:GbTCP4* were placed in nutrient soil (nutritional soil:vermiculite:perlite, 1:1:1). When the first two rosette leaves appeared, the number of leaf trichomes was counted. The stem trichomes on the main stem internodes were counted from the bottom of 1-month-old soil-grown seedlings. Images were collected under a SteREO Discovery.V20 microscope (Zeiss, Oberkochen, Germany). All experiments were repeated three times.

### 2.12. Phloroglucinol Staining

Thirty seedlings from three *35S:GbTCP4* transgenic *Arabidopsis* lines and WT *Arabidopsis* were planted in nutrient soil and used for phloroglucinol staining after 6 weeks of growth. Fresh stems of *Arabidopsis* were taken, and a 1 cm thick sample of the same part of the transgenic and WT stems was cut with a blade. A 1% phloroglucinol-alcohol solution was dropped on the cross-section of the sample and left for 2 min. The sample was dried with 32% concentrated hydrochloric acid solution until the sample cross-section appeared red. The sample staining results were observed with a Nikon Eclipse TS2R microscope (Nikon, Tokyo, Japan) and were imaged.

### 2.13. Determination of Lignin Content

The lignin content of the *35S:GbTCP4* transgenic *Arabidopsis* line and WT *Arabidopsis* stems was determined according to the procedure of the Plant Lignin ELISA Kit (Mlbio, Shanghai, China). All experiments were repeated three times.

### 2.14. Statistical Analyses

Statistical analysis of the numerical data was performed using GraphPad Prism 7.0 (GraphPad Software, San Diego, CA, USA) and SPSS 18.0 (SPSS Inc., Chicago, IL, USA). For multiple pairwise comparisons, the data were compared using a one-way ANOVA test. *p < 0.05 was considered statistically significant, and **p < 0.01 was considered extremely significant.

## 3. Results

### 3.1. Cloning and Sequence Analysis of GbTCP4

**Figure 1:**
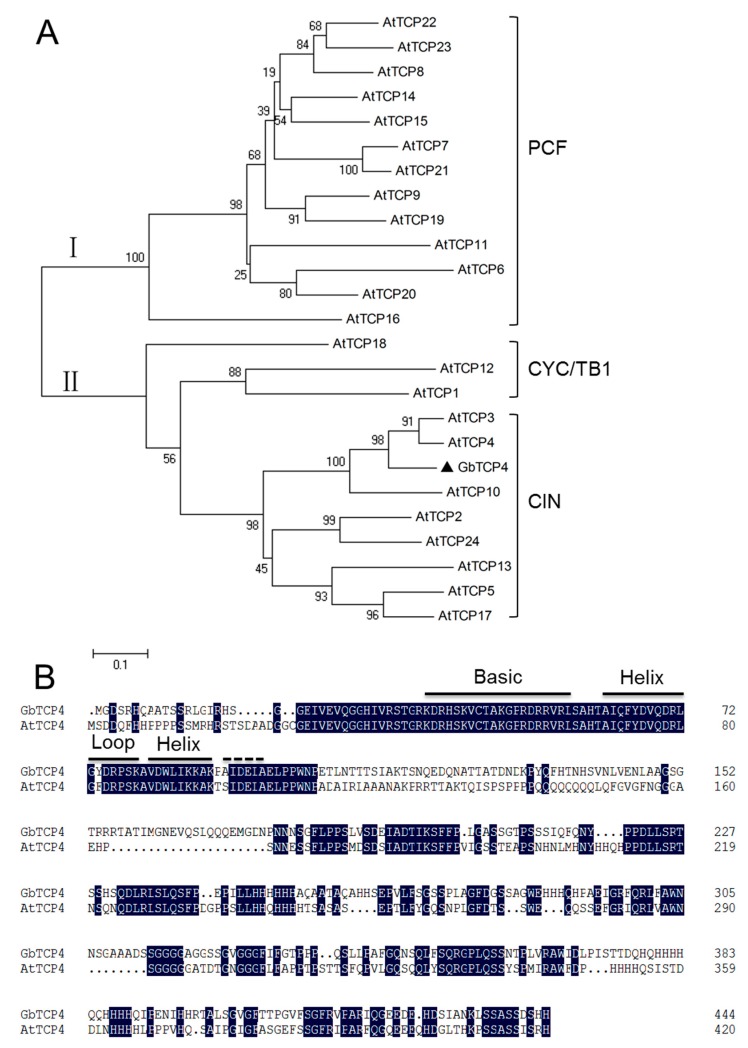
Phylogenetic tree analysis and sequence alignment of GbTCP4. (**A**) The phylogenetic tree of GbTCP4 and *Arabidopsis* TCP family proteins. The accession numbers of the proteins are as follows: AtTCP1, AT1G67260.1; AtTCP2, AT4G18390.1; AtTCP3, AT1G53230.1; AtTCP4, AT3G15030.1; AtTCP5, AT5G60970.1; AtTCP6, AT5G41030.1; AtTCP7, AT5G23280.1; AtTCP8, AT1G58100.1; AtTCP9, AT2G45680.1; AtTCP10, AT2G31070.1; AtTCP11, AT2G37000.1; AtTCP12, AT1G68800.1; AtTCP13, AT3G02150.2; AtTCP14, AT3G47620.1; AtTCP15, AT1G69690.1; AtTCP16, AT3G45150.1; AtTCP17, AT5G08070.1; AtTCP18, AT3G18550.1; AtTCP19, AT5G51910.1; AtTCP20, AT3G27010.1; AtTCP21, AT5G08330.1; AtTCP22, AT1G72010.1; AtTCP23, AT1G35560.1; AtTCP24, AT1G30210.1. (**B**) Comparison of amino acid sequences of GbTCP4 and AtTCP4. Black indicates identical amino acids. The horizontal line indicates the conserved teosinte branched1/cycloidea/proliferating cell factor1 (TCP) domain.

The *GbTCP4* gene was cloned from Xinhai 21 according to the cotton fiber transcriptome data of the sea-island cotton cultivar Xinhai 21. The ORF of *GbTCP4* is 1335 bp in length and encodes 444 amino acids with a predicted MW of 48.05 kDa and a pI of 7.16. The amino acid sequence of GbTCP4 contains a conserved TCP domain between 36 and 99 amino acids. Phylogenetic tree analysis found that GbTCP4 clustered with AtTCP3 and AtTCP4 in one branch, belonging to the CIN subclass of the class II TCP protein (Figure 1A). Multiple sequence alignment revealed that GbTCP4 shares 47.07% and 50.43% overall amino acid identity with AtTCP3 and AtTCP4, respectively. The amino acid sequence alignment of the TCP domain of GbTCP4 and AtTCP4 indicates that their amino acid identity is 95.31%, and there are only three different amino acids (Figure 1B). Moreover, it is predicted that there is a nuclear localization signal (PRDRRVR) in the basic structure of the TCP domain of GbTCP4.

### 3.2. GbTCP4 Protein Localizes to the Nucleus

**Figure 2:**
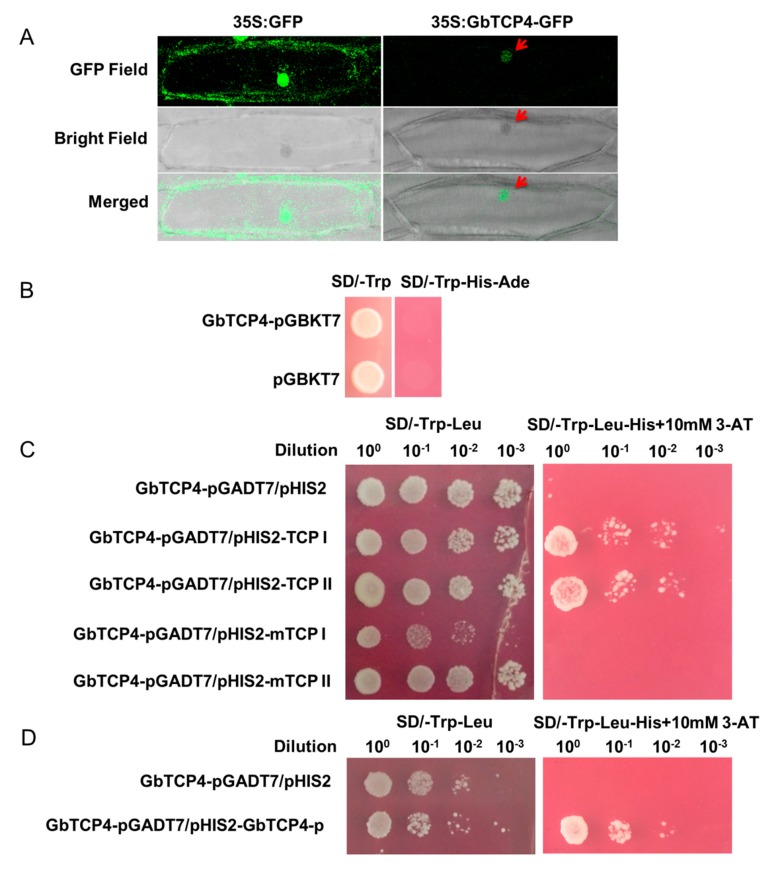
Molecular characterization of the GbTCP4 transcription factor. (**A**) Subcellular localization analysis of the GbTCP4 transcription factor. *35S:GFP* is an empty vector control. The red arrow indicates the nucleus. (**B**) Analysis of the transcriptional activation activity of GbTCP4. pGBKT7 is an empty vector control. (**C**) Analyses of the binding of GbTCP4 to TCP I (TGGGTCCCACAT), TCP II (TTGTGGGCCCCT), mTCP I (TAGATTCTAAAG), and mTCP II (TCGGGAGACTCG). (**D**) Analyses of the binding of GbTCP4 to the TCP-binding element in its promoter.

To analyze whether the GbTCP4 transcription factor is localized in the nucleus, the *Agrobacterium*-mediated method was used to transiently express *35S:GbTCP4-GFP* in onion epidermal cells, and cells transformed with *35S:GFP* served as a control. As shown in Figure 2A, compared with that transformed with *35S:GFP*, the onion epidermis transformed with *35S:GbTCP4-GFP* showed green fluorescence only in the nucleus. The subcellular localization results indicated that the GbTCP4 protein was localized to the nucleus.

### 3.3. GbTCP4 Lacks Transcriptional Activation Activity

To detect whether GbTCP4 is capable of activating the *HIS3* reporter gene in yeast, pGBKT7 and GbTCP4-pGBKT7 were transformed into AH109 yeast cells. Yeast cells transformed with pGBKT7 and GbTCP4-pGBKT7 grew normally on SD/-Trp medium (Figure 2B), indicating that both plasmids were transformed into yeast cells. However, yeast transformed with PGBKT7 and GbTCP4-pGBKT7 could not grow on SD/-Trp-His-Ade medium (Figure 2B). The above results indicate that GbTCP4 cannot activate the *HIS3* reporter gene alone in yeast and may need to activate or inhibit the target gene together with other transcription components.

### 3.4. GbTCP4 Binds to Two Types of TCP-Binding Cis-Elements

To examine whether GbTCP4 binds to the normal and mutated TCPI/II binding *cis*-element sequences, yeast one-hybrid analyses were employed. In SD/-Leu-Trp medium, all transformed yeast cells grew normally, indicating that the transformation was successful. However, in SD/-Leu-Trp-His 10 mM 3-AT medium, only the yeast cells co-transformed with pHIS2-TCP I/II and GbTCP4-pGADT7 grew normally, indicating that GbTCP4 recognizes two types of TCP-binding *cis*-elements (Figure 2C).

### 3.5. GbTCP4 Binds to TCP-Binding Cis-Elements in Its Promoter

Using the PLACE network to analyze the promoter sequence of *GbTCP4* [18], we identified five TCP-binding *cis*-acting elements in its promoter sequence (Supplementary Figure S1). Using the yeast one-hybrid method, as shown in Figure 2D, we determined that GbTCP4 binds directly to TCP-binding *cis*-elements in its promoter.

### 3.6. Expression Pattern of GbTCP4 in Cotton

**Figure 3:**
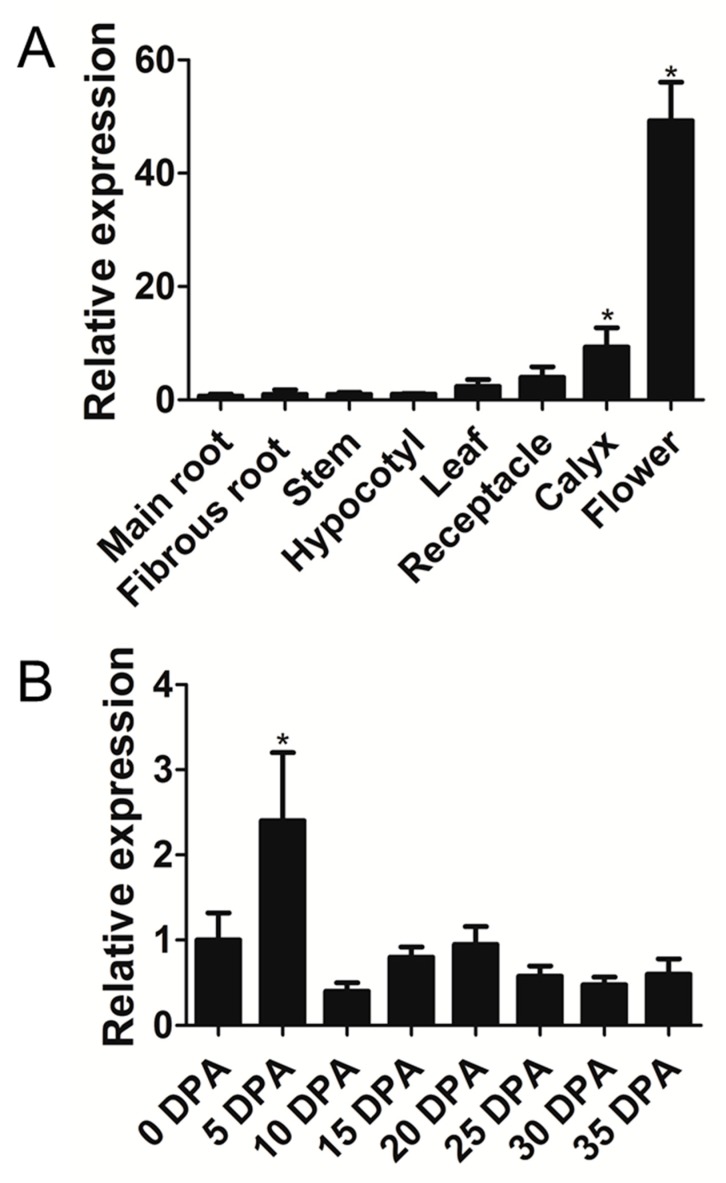
The expression pattern of the *GbTCP4* gene in different tissues and fibers at different developmental stages of cotton. (**A**) Transcriptional levels of *GbTCP4* in the cotton main root, fibrous root, stem, hypocotyl, leaf, receptacle, calyx, and flower. The transcriptional levels were calibrated by the control (hypocotyl) (**B**) Transcriptional levels of *GbTCP4* in developing cotton fibers collected at 0 (ovule), 5, 10, 15, 20, and 25 d post anthesis (DPA). The transcriptional levels were calibrated by the control (0 d days post anthesis (DPA)). *GbUBQ7* is used as an internal reference gene. Values represent the means of three biological replicates, and error bars represent standard deviations. Asterisks indicate a significant difference (*p < 0.05) relative to the corresponding controls.

qPCR was used to analyze the expression pattern of *GbTCP4* in different tissues and organs of cotton. The qPCR results showed that the transcript level of *GbTCP4* was the highest in flowers, while its transcript levels were lower in calyx, receptacle, leaf, hypocotyl, stem, and fibrous root tissues, and the lowest transcript level occurred in the main root (Figure 3A). qPCR showed that *GbTCP4* was expressed in cotton fibers at different developmental stages, and it had the highest transcript level in cotton fibers at 5 days post anthesis (DPA), suggesting that GbTCP4 may be involved in regulating the elongation of cotton fibers (Figure 3B).

### 3.7. Overexpression of GbTCP4 Increases Root Hair Length and Density

**Figure 4:**
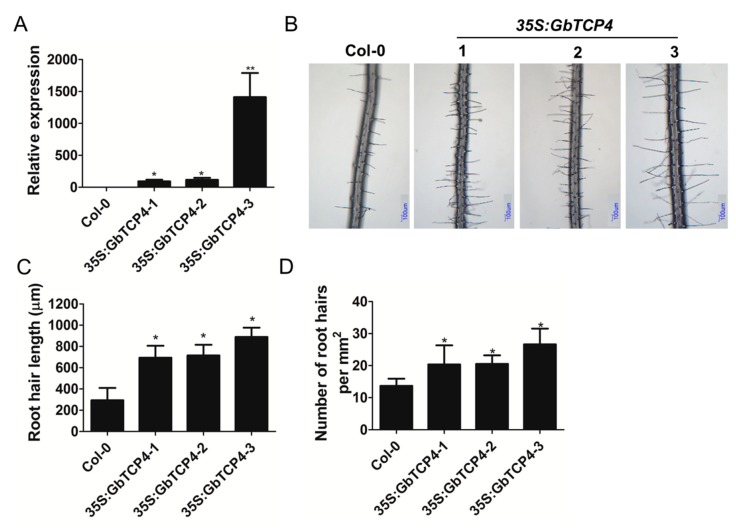
Constitutive overexpression of *GbTCP4* in *Arabidopsis* increases root hair length. (**A**) Transcriptional levels of the *GbTCP4* gene in three *35S:GbTCP4* transgenic *Arabidopsis* lines. The transcriptional levels were calibrated by the control (Col-0). *AtUBQ3* was used as an internal reference gene. Values represent the means of three biological replicates, and error bars represent standard deviations. (**B**) Root hair phenotype from 7-d-old vertically grown seedlings of Col-0 and *35S:GbTCP4* transgenic lines. Scale bars: 100 µm. (**C**) Comparison of the root hair length on the roots from the Col-0 and *35S:GbTCP4* transgenic lines. (**D**) Comparison of the root hair density on the roots from the Col-0 and *35S:GbTCP4* transgenic lines. Values are means (±SE) of 30 seedlings for each of three independent experiments. Asterisks indicate significant differences (*p < 0.05; **p < 0.01) relative to Col-0.

To reveal the biological function of *GbTCP4*, it was overexpressed in *Arabidopsis* plants. We selected three representative homozygous transgenic plants for functional analysis. The *GbTCP4* gene had a very high transcript level in the three homozygous *35S:GbTCP4* transgenic lines, whereas no transcript level expression of the *GbTCP4* gene was detected in WT plants (Figure 4A). Because *GbTCP4* has a high transcript level during cotton fiber elongation, we examined its effect on *Arabidopsis* root hair development. *Arabidopsis* plants with two true leaves were placed in plates with MS medium for vertical culture. Approximately 7 d after vertical growth, compared with that of WT plants, the root hair length of the primary roots of three *35S:GbTCP4* transgenic lines significantly increased; the root hair lengths of the three *35S:GbTCP4* transgenic lines were 2.37, 2.44, and 3.03 times the WT root hair length, respectively (Figure 4B,C; Supplementary Table S2). Moreover, compared with that of WT plants, the root hair density of *35S:GbTCP4* transgenic plants significantly increased (Figure 4D; Supplementary Table S2). The above results suggest that the overexpression of *GbTCP4* increases root hair length and density in the primary roots of *Arabidopsis*.

### 3.8. Overexpression of GbTCP4 Increases the Trichome Number of the Leaf and Stem

**Figure 5:**
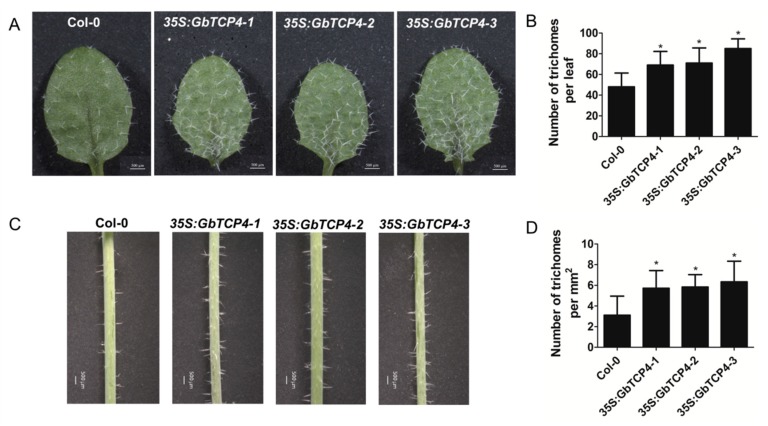
Constitutive overexpression of *GbTCP4* in *Arabidopsis* affects trichome formation. (**A**) Leaf trichome phenotype from the first two rosette leaves of 2-week-old soil-grown seedlings of the Col-0 and *35S:GbTCP4* transgenic lines. Scale bars: 500 µm. (**B**) Comparison of the trichome number on the first two rosette leaves from Col-0 and *35S:GbTCP4* transgenic lines. (**C**) Stem trichome phenotype from the main stem internodes from the bottom of 1-month-old soil-grown seedlings of Col-0 and *35S:GbTCP4* transgenic lines. Scale bars: 500 µm. (**D**) Comparison of the trichome density on the main stem internodes from the bottom of Col-0 and *35S:GbTCP4* transgenic lines. Values are means (±SE) of 30 seedlings for each of three independent experiments. Asterisks indicate significant differences (*p < 0.05) relative to Col-0.

To study whether GbTCP4 regulates trichome formation, trichomes on the leaves and stems of WT and *35S:GbTCP4* transgenic plants were observed. Statistical analyses showed that, compared with WT plants, the three *35S:GbTCP4* transgenic lines had significantly increased trichome numbers on the first two rosette leaves, and the trichome numbers of the three *35S:GbTCP4* transgenic lines were 1.44, 1.48, and 1.77 times those of the WT plants, respectively (Figure 5A,B). Similarly, the trichome numbers on the main stem internodes from the bottom of three *35S:GbTCP4* transgenic lines were significantly higher than those of WT plants (Figure 5C,D). These results indicate that overexpression of the *GbTCP4* gene in *Arabidopsis* increases the trichome number of leaves and stems.

### 3.9. Overexpression of the GbTCP4 Gene Increases Lignin Content in Transgenic Arabidopsis

**Figure 6:**
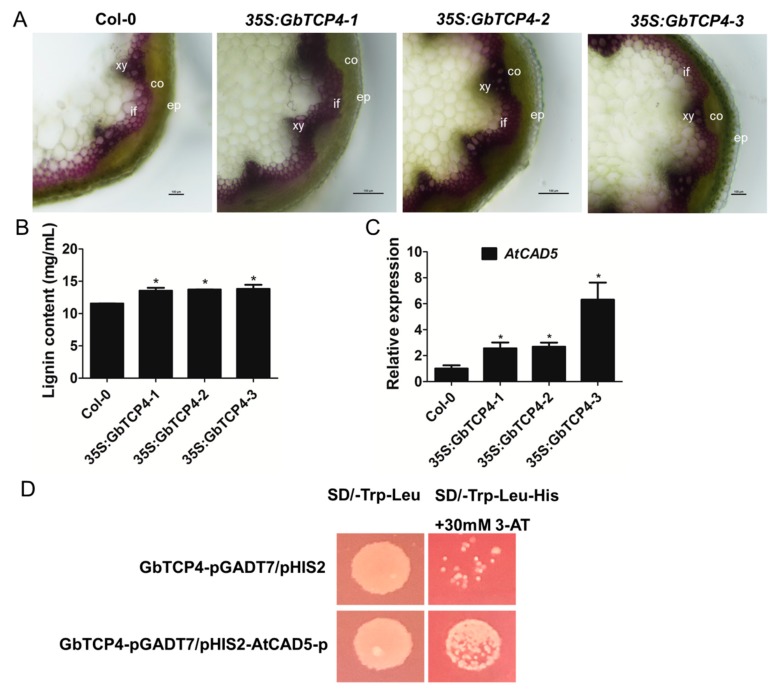
Constitutive overexpression of the *GbTCP4* gene increases lignin content in transgenic *Arabidopsis* stems. (**A**) Phloroglucinol staining of stem cross-sections of the Col-0 and *35S:GbTCP4* transgenic lines. xy: xylem; if: interfascicular fibers; co: cortex; ep: epidermis; bars: 100 µm. (**B**) Determination of lignin content in stems of the Col-0 and *35S:GbTCP4* transgenic lines. (**C**) Quantitative PCR (qPCR) analysis of transcriptional levels of the *AtCAD5* gene in the Col-0 and *35S:GbTCP4* transgenic lines. The transcriptional levels were calibrated by the control (Col-0). *AtUBQ3* was used as an internal reference gene. Values represent the means of three biological replicates, and error bars represent standard deviations. Asterisks indicate significant differences (*p < 0.05) relative to Col-0. The accession number of the gene is as follows: AtCAD5:At4g34230. (**D**) Analyses of the binding of GbTCP4 to the TCP-binding *cis*-element in the promoter of *AtCAD5*.

Previous studies have found that overexpression of *AtTCP4*, a homologue of *GbTCP4*, increases the lignin content of transgenic *Arabidopsis* [21]. To analyze whether GbTCP4 is involved in the regulation of lignin content, lignin staining was performed on the *35S:GbTCP4* transgenic line and WT *Arabidopsis* using the phloroglucinol method. As shown in Figure 6A, the cross-sections of the three *35S:GbTCP4* transgenic line stems were darker, and the red color was denser than that observed for the WT plants, indicating that the three *35S:GbTCP4* transgenic lines had higher lignin content. The lignin content of the stems showed that the lignin content of the three *35S:GbTCP4* transgenic lines was significantly higher than that of the WT plants. Compared with that of WT plants, the lignin content of the three *35S:GbTCP4* transgenic lines increased by 17.13%, 18.77%, and 19.64%, respectively (Figure 6B). Cinnamyl alcohol dehydrogenase (CAD; EC 1.1.1.195) catalyzes the final step in lignin precursor synthesis, reducing the cinnamyl aldehydes to their corresponding alcohols in the presence of NADPH [22]. Subsequently, qPCR was used to analyze the transcriptional level of the *AtCAD5* gene in the three *35S:GbTCP4* transgenic *Arabidopsis* lines. The results showed that the transcript level of *AtCAD5* was significantly elevated in the three *35S:GbTCP4* transgenic line relative to the WT plants (Figure 6C). In addition, analyses of *cis*-acting elements in the promoter revealed TCP-binding elements in the promoter of *AtCAD5*. Yeast one-hybrid results indicated that GbTCP4 can bind the TCP-binding elements in the *AtCAD5* promoter (Figure 6D). These results indicate that overexpression of the *GbTCP4* gene increases the lignin content of the stem of transgenic *A. thaliana*. Moreover, *AtCAD5* increased transcript levels in the *35S:GbTCP4* transgenic *Arabidopsis* line.

### 3.10. CPC is a Target Gene of GbTCP4

**Figure 7:**
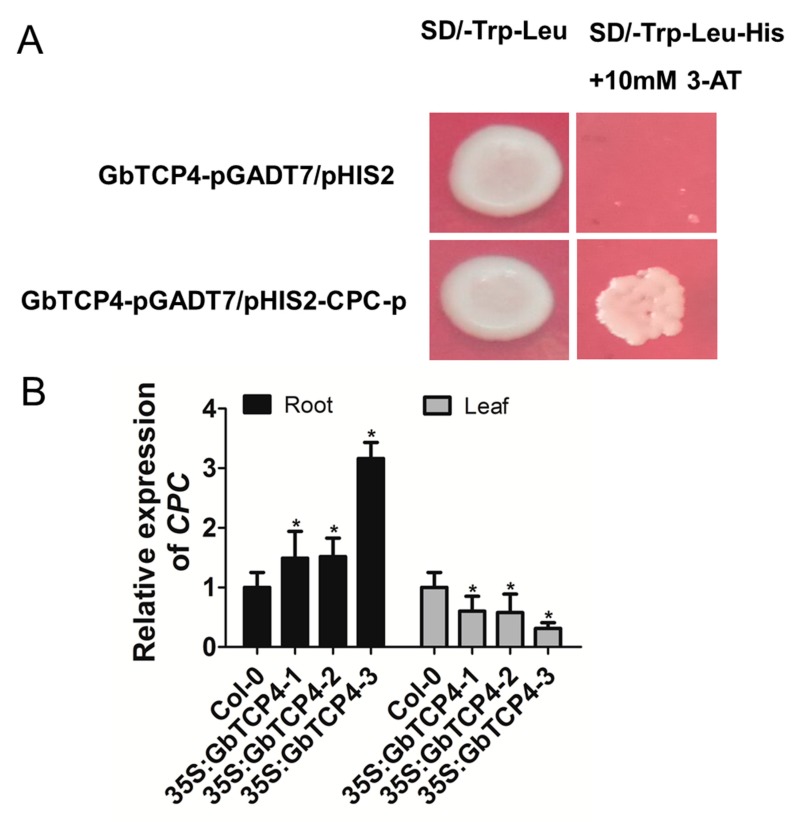
*CAPRICE* (*CPC*) is a target gene of *GbTCP4*. (**A**) Analyses of the binding of GbTCP4 to the TCP-binding *cis*-element in the promoter of *CPC*. (**B**) Detection of the transcription levels of *CPC* gene in the roots and leaves of Col-0 and *35S:GbTCP4* seedlings by qPCR. The transcriptional levels were calibrated by the control (Col-0). *AtUBQ3* expression was used as a control. Values represent the means of three biological replicates, and error bars represent standard deviations. Asterisks indicate significant differences (*p < 0.05) relative to Col-0.

To identify the target gene of *GbTCP4*, the PLACE network was used to analyze whether promoter regions of some of the known transcription factor genes involved in root hair and trichome development contained TCP-binding elements [18]. Analyses of *cis*-acting elements in the promoter revealed TCP-binding elements in the promoter of *CPC*. Yeast one-hybrid results indicated that GbTCP4 can bind the TCP-binding elements in the *CPC* promoter (Figure 7A). Then, the transcript level of the *CPC* gene in the roots and leaves of the three *35S:GbTCP4* transgenic line were analyzed by qPCR. qPCR results revealed that, compared with that of WT plants, the transcript level of *CPC* in the roots of the three *35S:GbTCP4* transgenic line were elevated, and the transcript level of *CPC* in the leaves were reduced (Figure 7B). These results indicate that *CPC* is a target gene of *GbTCP4.*

## 4. Discussion

### 4.1. GbTCP4 Regulates Root Hair Length and Trichome Number in Arabidopsis

Previous research has found that some *TCP* genes are highly expressed in cotton fibers [11,13]. For example, the transcriptome data and qPCR results showed that 15, 9, and 12 *GhTCP* genes from upland cotton were specifically expressed in the fiber initiation, elongation, and secondary cell wall deposition stages, respectively [13]. Similarly, 13 of the 36 *G. arboreum* TCP transcription factors are highly expressed at different fiber development stages [11]. Our present research also found that the *GbTCP4* gene had a higher transcript level in rapidly elongating fibers, which suggests that it is involved in cotton fiber elongation. Because *Arabidopsis* leaf trichomes have been successfully used as an ideal model research system for the functional analyses of cotton fiber development-related genes [14,15], we constitutively overexpressed *GbTCP4* in *Arabidopsis* to study its function in trichome development. We found that, compared with those of WT plants, the root hair length and trichome numbers of plants overexpressing *GbTCP4* increased. These results strongly show that GbTCP4 regulates root hair length and trichome number in *Arabidopsis*.

### 4.2. Mechanism of the Action of GbTCP4 in the Regulation of Root Hair Length and Trichome Number in Arabidopsis

Trichome initiation and patterning of *Arabidopsis* are activated by a MYB-bHLH-WD40 complex comprising three positive regulators (GL1, GL3, and TTG1) [23]. Conversely, in *Arabidopsis*, an R3-type MYB domain protein, AtCPC, negatively regulates trichome initiation and patterning. *Arabidopsis* overexpressing *AtCPC* has reduced or no trichomes on the leaves and more root hairs on the roots [24]. Moreover, TTG1 represses the activation of the CPC promoter by GL1 and GL3 [25]. Similarly, in cotton, *GhTTG1* and *GhTTG3* restore trichome formation in *ttg1*-mutant *Arabidopsis* plants [26]. *GhCPC* is homologous to *AtCPC*, and transgenic cotton lines overexpressing *GhCPC* showed delayed fiber initiation and reduced fiber length by a potential CPC-MYC1-TTG1/4 complex [27]. GhTCP22 and GhTCP14a can interact with GhMYB25/GhMYB23-GhGL3-GhTTG1 [13]. Our results showed that overexpression of *GbTCP4* in *Arabidopsis* can activate the transcript levels of *CPC* in roots and inhibit the transcript levels of CPC in leaves. This may be because some of the TCP proteins, including the *GbTCP4* protein, do not enable activation or inhibition by themselves and need to cooperate with other proteins to regulate the transcription of the target gene. Previous studies have found that two class I proteins, GhTCP22 and GhTCP14a, can interact with several transcription factors related to cotton fiber growth and development. Both can interact with GhSLR1, GhGL3, GhARF6, GhTTG1, GhMYB23, and GhMYB25 transcription factors. In addition, GhTCP14a can also interact with GhEIN3, GhBZR1, and GhMYB25L proteins [13]. Because cotton fiber development is similar to that of *Arabidopsis* trichome development, we speculate that the *GbTCP4* gene may promote fiber elongation in cotton through a similar regulatory mechanism.

### 4.3. Overexpression of GbTCP4 Increases Lignin Content

Previous studies found that, when inhibition of *AtTCP4* by *miR319a* was abolished, petals and stamens did not develop [28]. The *AtTCP4* promoter drives the *GUS* reporter gene to be expressed in petals, unflowered pollen and carpels [29]. Similarly, we found that *GbTCP4* has a high level of transcriptional expression in the flower, calyx, and receptacle of sea-island cotton. Recent studies have found that overexpression of *miR319*-resistant *AtTCP4* increases the lignin and cellulose content of transgenic *Arabidopsis* [21]. Similar to this result, we found that, compared with WT plants, transgenic *Arabidopsis* plants that overexpress the *GbTCP4* gene exhibit increased lignin content. Moreover, *AtCAD5* increased transcript levels in the *35S:GbTCP4-3* transgenic *Arabidopsis* line. AtCAD5 was capable of efficiently catalyzing all five substrates, including p-coumaryl aldehyde, caffeyl aldehyde, coniferyl aldehyde, 5-hydroxyconiferyl aldehyde, and sinapyl aldehyde. AtCAD5 can be used to normally generate all three monolignols. Mutation of the *AtCAD5* gene slightly delayed the syringyl lignin deposition rate [22]. We speculate that GbTCP4 may be involved in the regulation of *AtCAD5* gene expression and thus may affect lignin content.

### 4.4. GbTCP4 Recognizes Two Types of TCP-Binding Cis-Elements

The TCP-binding *cis*-element sequences of the two classes are different but overlapping, but some TCP proteins show certain adaptability to recognition [8]. For instance, the class I transcription factor AtTCP16 can bind to TCP II binding sites [30]. In cotton, GhTCP14, a class I TCP transcription factor, can bind to TCP I and II binding sites [15]. Similarly, our results showed that GbTCP4, as a class II TCP transcription factor, could also bind to TCP I and II motifs. Previous research has shown that two classes of TCP proteins can bind to CG-rich sequences in target gene promoters, and speculations exist about a potential opposing relationship between class I and class II TCPs [8]. For example, GhTCP14 can recognize the promoters of PIN2, IAA3, and AUX1 [15]. Our results showed that GbTCP4 binds to the promoter of *AtCPC* and regulates its expression.

## 5. Conclusions

In conclusion, GbTCP4 acts as a transcription factor, localizes to the nucleus, and is capable of binding two types of TCP-binding cis-acting elements. GbTCP4 is involved in the regulation of *Arabidopsis* root hair and trichome development and lignin deposition and regulates the transcript levels of related genes. Our results not only increased understanding of the function of the sea-island cotton class II TCP protein but also provided candidate genes for improving cotton fiber length.

## Supplementary Materials

The following are available online at https://www.mdpi.com/2073-4425/10/9/726/s1, Figure S1: The promoter sequences of *GbTCP4*. The predicted TCP-binding site is represented by yellow, Table S1: Primer sequences used in this study, Table S2: Data for measuring the number and length of root hair.
