# Supplementary material for: Heterologous Expression of GbTCP4, a Class II TCP Transcription Factor, Regulates Trichome Formation and Root Hair Development in Arabidopsis
Source: Genes (Basel). 2019 Sep 19;10(9):726. doi: 10.3390/genes10090726 (PMC6771151; doi:10.3390/genes10090726)
Supplement: Supplementary file 1 [file genes-10-00726-s001.zip › Table S1 and Figure S1.pdf]

Table S1. Primer sequences used in this study.

Name	Sequence
TCP-I-F	5'-
	AATTAGTCCTGGGTCCCACATGCCTGGGTCCCACATGCCTGGGTCC
TCP-I-R	CACATGCTGTTCTCGAAGCT-3'
	5'-
TCP-II-F	TCGAGAACAGCATGTGGGACCCAGGCATGTGGGACCCAGGCATGT
	GGGACCCAGGACT-3'
TCP-II-R	5'-
	AATTAGTCCTTGTGGGCCCCTGCCTTGTGGGCCCCTGCCTTGTGGG
TCP-mI-F	CCCCTGCTGTTCTCGAAGCT-3'
	5'-
TCP-mI-R	TCGAGAACAGCAGGGGCCCACAAGGCAGGGGCCCACAAGGCAGG
	GGCCCACAAGGACT-3'
TCP-mII-F	5'-
	AATTAGTCCTAGATTCTAAAGGCCTAGATTCTAAAGGCCTAGATTC
TCP-mII-R	TAAAGGCTGTTCTCGAAGCT-3'
	5'-
CPC-PH-F	TCGAGAACAGCCTTTAGAATCTAGGCCTTTAGAATCTAGGCCTTTA
CPC-PH-R	GAATCTAGGACT-3'
pHIS2-F	5'-
pHIS2-R	AATTAGTCCTCGGGAGACTCGGCCTCGGGAGACTCGGCCTCGGGA
nGbTCP4-F	GACTCGGCTGTTCTCGAAGCT-3'
nGbTCP4-R	5'-
GbTCP4-qF:5	TCGAGAACAGCCGAGTCTCCCGAGGCCGAGTCTCCCGAGGCCGAG
GbTCP4-qR:5	TCTCCCGAGGACT-3'
pGADT7-GbTCP4-F	5'-CCGGAATTCGACCACGTGAACCCATT-3'
pGADT7-GbTCP4-R	5'-CGAGCTCGTCAGCATCTGTATGCCA-3'
3301-GbTCP4-F	5'-AGTCACGACGTTGTAAAACGACG-3'
3301-GbTCP4-R	5'-ATCTTTGCCTTCGTTTATCTTGCCT-3'

1304-GbTCP4-F	5'-ATGGGAGATAGCCGCCACCAAGCTG-3'
1304-GbTCP4-R	5'-TCAATGGTGAGAATCAGATGAAGCAGAGG-3'
P4-PH1-F	5'-AGGAGGTCACATTGTTCGGTCA-3'
P4-PH1-R	5'-TCGTAGCCAAGTCGGTCCTG-3'
CPC-F	5'-CGCATATGATGGGAGATAGCCGCCACCAAGCTG -3'
CPC-R	5'-CCCCCGGGTCAATGGTGAGAATCAGATGA-3'
AtUBQ3-F	5'-CCCATGGGATGGGAGATAGCCGCCACCAAGCTG -3'
AtUBQ3-R	5'-CGCACGTGCGTCAATGGTGAGAATCAGATGA-3'
pGBKT7-GbTCP4-F	5'-CCCCGGGG ATGGGAGATAGCCGCCACCAAGCTG-3'
pGBKT7-GbTCP4-R	5'-CGGGATCCCGATGGTGAGAATCAGATGA-3'
GbUBQ7-F	5'-CCGGAATTCTTGGCAGGCTTGGTTAGG-3'
GbUBQ7-R	5'-CGAGCTCAGCCTCTGCCATTCAATG-3'
CAD5-F	5'-ATGTTTCGTTCAGACAAGGC-3'
CAD5-R	5'-TCATTTCCTAAAAAAGTCCT-3'
	5'-CGGAAAGACCATTACTCTGGA-3'
	5'-CAAGTGTGCGACCATCCTCAA-3'
	5'-CCCATGGG ATGGGAGATAGCCGCCACCAAGCTG -3'
	5'-CGCACGTGCGCAATGGTGAGAATCAGATGA-3'
	5'-GACCTACACCAAGCCCAAGAAG-3'
	5'-TGAGCCCACACTTACCACAATAGT-3'
	5'-TTGGCTGATTCGTTGGATTA-3'
	5'-ATCACTTTCCTCCCAAGCAT-3'

AAATAAAGCATAAAAATAAATATTTTTTTAAAAAAAGCTGTGAATTGGGGTCACTGTCAAAAGTTGTGATGGT
GGCATATAAAGAAAGGATCTATTCATTCATCTGATCTAATCAAAACAAAGCATCCAGACAAATCCCTTCAACTA
TAACCAACACTTCTTAACTTCTAGCTATGGAACTAAGTTAAAAAAAAAGAAAAAAGACTGAAAAGGTTGTTA
CTTTCTTACCACTTACTAGGTTATCAATTTTTAAGACAGGTTGAAAATTTAAAGTCATGTAAATCAAATCTATCT
ATACCTTAGTAAAAAATAAGAAAAAGGGTTATATAATTTATACTGGAAATGAGAAAAGTTGAAAAAAAAAAG
GGTGGATTTTAGGTTGGTTGAAATGGGGAGAGAGGTGGGAAAAAATGGTGTTTTAGATTTGTGTAGTTTTA
GGGTAAAAGTACAGAATAAAACAGCAGCAAATGCAGTGATGAGATGAACAGAGATCTTTAATCTTTTGTCTG
ACCCCAAAAACACTGCCACCCTTAGCAGTGGCATCATCATCAACATCAAGGGTGTTTCTATAGGCCAAAGTA
AAGTGTGTTTACTCTTCCCTATACTATAGCTATTAAGCTATAAGCCCTATATATATTTTGCTCTTCTAAACAAACC
AAGTTTTTTTTCTCTCTTTTCGTTTAGTTGTAGCTTGTCATGAAAGAGTTTCTAGTTAGTATAGTTTCCCAAGA
AAATCCTTGTTGAGAAGAGAACATTTTGTTTTTGTTGTTGTTGGTGGTGGGTGGCTAAGTGTTTGAAAACCC
GTTTCGTTGCTGAATAGAATAGTACATAAATAACAGAGAGCTCCTATATTGCCAAAAGCTGAGTTTTTTAGAG
AGAAAATAGAAAGAGAAACAAGGAGAAAAAAACGATGGGTTAGGCTGGGGGAATAATAGGGGCTGGGGT
TTTTTTGTTTTTTTGTTTTAGTTGGCAGGCTTGGTTAGGGAAGAGTGGTGGTGGTGGTGGGTGGGGGGGC
CGGCCGTTTGGTGTGTGTGGTATGATGGAATTAGAATCATTGAATGGCAGAGGCTTTTGAGAGAGGTCACA
GTATCAGGTTTCAGTGTGGTGGCAGTAGCTCATACAGGTAGAGGATATTAGCAAGCAAAGAACAACAACTAC
AACCCAGTAACATAAAAAAGGTATCCTCCATTAAAAGCTGGAACAGCAAAAGCACCAGGGCCTGACTCTGG
ATCCTCAAAGGCATCATCATCATCAGCAGCAGCAGCAGCAGCAGCAGCAACAGCAACATCAGAATCCATCTC
ATATAGGAGGAGGAGGAGAAGAAGAAGAAATTGCGAGGAACCAACAGCAAATCTTGTTTCTCCATCATCAA
CAACAGCAAGGGCAACAGTTGTATGCAAGCCTGGAAACTCAAGTACTACGAGAGCAGCAAGGGCAACAAC
AACAGCAAGAGTACCCACAACCACCCAAGAAACGCTCTTATCTGCCGTCGGCTTCTACTTCAACTTTTGGTT
CCAAATCAACCCAACATGCAAGAAAA

Figure S1. The promoter sequences of *GbTCP4*. The predicted TCP-binding site is represented by yellow.
